# Is it time to make brain health a vital sign?

**DOI:** 10.1016/j.tjpad.2026.100654

**Published:** 2026-08-13

**Authors:** Gregory Cooper

**Affiliations:** Norton Healthcare, Kentucky, United States

Alzheimer’s Disease and related disorders are among the leading causes of disability worldwide. It is estimated that over 55 million people are currently living with dementia across the world, with that number projected to grow to 139 million by 2050 [1]. While the advent of amyloid targeting therapies (ATT’s) in Alzheimer’s Disease has represented a significant step forward, treatment options remain limited. Additionally, these therapies are only indicated for patients with mild cognitive impairment (MCI) due to Alzheimer’s Disease or mild Alzheimer’s Disease, leaving many patients ineligible. While research continues to explore various other pharmacologic strategies for the treatment of dementia, the role of lifestyle has begun to emerge.

Interest in brain health has grown steadily in recent years, likely in part related to the public’s fear of cognitive decline. Dementia has consistently been listed as a leading health concern among older adults. The Lancet Commission has provided periodic updates listing modifiable risk factors, currently numbered at 14, and accounting for approximately 45% of the risk of dementia worldwide. A number of other organizations, including the American Heart Association, the Alzheimer’s Association and the World Health Organization have provided their own lists of risk factors and recommendations for healthy lifestyles. Though not confined to cognitive health, the American Academy of Neurology has made Brain Health a cornerstone of its mission. Building on this we are now increasingly seeing the results of large clinical trials testing the impact of lifestyle interventions on cognition and risk of decline.

The landmark Finnish Geriatric Intervention Study to Prevent Cognitive Impairment and Disability (FINGER), published in 2015, established the ability of a lifestyle intervention to preserve and even improve cognition, setting the stage for subsequent studies. This randomized controlled trial examined the utility of a multi-domain lifestyle intervention in an at-risk elderly population. The intervention included diet, exercise, cognitive training, social engagement and vascular risk factor monitoring in patients with normal or slightly lower than expected cognition for age [2]. The authors concluded that the multi-domain intervention could improve or maintain cognitive function in elderly individuals at risk for dementia. This was followed 10 years later by the US POINTER study [3], intended to replicate the findings of FINGER in a diverse US population. Patients were randomized to a guided program of exercise, diet, cognitive challenge, social engagement and cardiovascular risk factor monitoring versus education and self-guided intervention. Interestingly both groups showed improvement over baseline, though those in the guided arm showed a statistically significant greater improvement in global cognition. This has since been followed by the LatAm-FINGERS study published in 2026 [4], showing again improvements in cognition associated with a multi-domain lifestyle intervention, this time in an ethnically diverse population across Latin America.

While these large studies have primarily focused on the role of lifestyle in risk reduction, there is some initial evidence and a growing consensus supporting the use of similar strategies for those with Mild Cognitive Impairment (MCI) and early dementia. However, little data currently exists regarding the clinical implementation of lifestyle strategies in the management of MCI and early dementia.

Against this backdrop comes the welcome report from Daffner et al [5] published in this edition of the Journal, *Suboptimal adherence to brain health recommendations in patients pursuing anti-amyloid antibody therapy for Alzheimer’s disease: Report from the Brain Health Vital Signs project*. The authors created a Brain Health Vital Signs (BHVS) score based on a series of 15 lifestyle risk factors, with individual scores ranging from 0-2 based on level of adherence. They are now reporting baseline results across two clinic populations at their institution. Strengths of the BHVS include relative ease of use for most elements and the ability to track changes over time. However, it is noted that some elements required extraction of laboratory data from the electronic health record which is likely to be more cumbersome in many clinical settings.

The authors calculated BHVS scores both among patients in their amyloid-targeting therapy (ATT) clinic and patients in a separate cognitive neurology subspecialty clinic. While these two groups are not perfectly comparable, with the later group including non-AD dementias, the results obtained are compelling. The authors hypothesized that those pursuing ATT’s would be more motivated and therefore show a higher level of compliance with a brain healthy lifestyle. Instead, they found relatively low levels of compliance across both groups, with <3% of patients following recommendations for all 15 items in the BHVS. Many potential reasons for low adherence were provided, including lack of knowledge, poor motivation, time constraints, financial limitations and sub-optimal medical management. Interestingly, cognitive impairment was not felt to be a significant factor based on the lack of difference in adherence between those with MCI and those with mild dementia. Rather, poor adherence was speculated to represent longstanding habits pre-dating the onset of cognitive impairment. And it is noted that as these lifestyle factors are known risk factors for dementia, relatively poor compliance at baseline is perhaps not surprising.

One could also speculate that this represents a failure of our current systems. Our specialty clinics currently are largely focused on the evaluation and diagnosis of patients with cognitive decline, and then to institution of pharmacologic interventions. Much work has gone into the creation of systems to administer ATT’s. However, perhaps less has been done to create the necessary systems to address lifestyle. While the low adherence rates found by Daffner et al [5] may be discouraging they may not be surprising, and they help provide a baseline for future study. We now need to develop and study interventions to improve adherence, which can potentially be measured as change in the BHVS or other similar measures. Potential strategies might include more robust use of patient navigation, patient and community outreach and educational programming, and stronger partnerships with primary care. For healthcare systems engaged in value-based care, the authors correctly point out that “it may be time for them to increase their investment in developing more effective, system-wide strategies and tangible, individualized interventions for addressing modifiable risk factors for cognitive decline and dementia for all the patients under their care.” This may herald a paradigm shift toward prevention, in this case including tertiary prevention, which will require us to create new models of care. The data we receive in the future from Brain Health Vital Signs project will be eagerly awaited.

## Authorship/CRediT authorship contribution statement

G.C. was responsible for the conceptualization and all writing of this editorial.

G.C.’s institution receives research support from Eisai, Novartis and Lilly.

Generative AI and AI-assisted technologies were not used in the preparation of this editorial.

## Declaration of competing interest

The authors declare that they have no known competing financial interests or personal relationships that could have appeared to influence the work reported in this paper.
